# Book Review

**Published:** 1915-03

**Authors:** 


### Reviews of Books.

---

**Sexual Ethics.** By ROBERT MICHELS. Pp. xv, 296. London: The Walter Scott Publishing Co. Ltd. Price 6s. net.—This book is purposed for the laity rather than the medical profession, and is written in a way to be understood of all. It consists, to use the author's words, of "empirical observations, psychological and sociological in character. Its issue presupposes, as is now the wont, that there are complex problems of sexual life needing to be fathomed, a mysteriously profound relationship between the sexes to be still further elucidated, and a constant and prejudicial reverie on procreative function to possess the minds of the rising generation. Be it said that the author, though expressing himself with frankness, has not willingly pandered to any would-be salacious palate, and although there is the usual review of prostitution and illicit intercourse in general, of the hygiene of matrimonial life and its transgression, of monogamy and polygamy, and so forth, we do not find anything which need bring a blush to the cheek of the not too young person. We would not recommend it, however, to any but those of mature years. We think the author's advice as to the gradual enlightenment of children in sexual matters the best that can be given under the vexed circumstances ; he especially deprecates the mystification of the curious young by fables or directly false statements, or even the constant shelving of all instruction until a full revelation be forced at puberty. It will be seen, nevertheless, in his very impartial treatment of the subject, that the treatment of the child must be individual, and, far preferably, the enlightenment should be made by the parents. Dr. Michels candidly avows and justifies his approval of the reasonable limitation of families by preventive measures, and pleads that means should be devised to render these less offensive to delicate susceptibilities and more certain in achieving their object. His suggestions to married women as to methods for preserving conjugal fidelity are naive and no doubt well based : we will not quote them here. It cannot be said that there is anything very novel in this work—the whole subject has been discussed at all times and in all places almost *ad nauseam ;* but there is doubtless a considerable section of the lay public who are interested in these matters, and who have escaped the mass of often offensive sex literature launched on the medical reviewer. To these, provided they do not seek merely prurient matter, we can recommend Dr. Michel's book. It bears the stamp of honest purpose and measured expression, and is worthy of the contemporary science series which has issued it.

**A Handbook for the Post-Mortem Room.** By ALEXANDER G. GIBSON, D.M., F.R.C.P. Pp. viii, 140. London : Oxford Medical Publications. 1914. Price 3s. 6d. net.—This manual fulfils its purpose admirably, and contains within a brief space all the detailed instruction necessary for the *post-mortem* clerk or demonstrator. It will be found accurate in detail and very practical, and should be widely used by students and resident officers. It is perhaps too elaborate for those who only very occasionally have the opportunity of making an autopsy. The Basle nomenclature is adopted throughout.

**Spectrum Analysis Applied to Biology and Medicine.** By the late C. A. MACMUNN, M.A., M.D. Pp. xiv. 112. London: Longmans, Green & Co. 1914. Price 5s.—This little book is prefaced by a graceful personal tribute to the late author by Prof Gamble, who calls attention to the fact that the research was carried out when he was far removed from the facilities of a laboratory or university, and in the scanty leisure afforded by a practice and volunteer duties. The book gives an account of MacMunn's valuable observations on the spectra of the various pigments of plants and invertebrates, as well as those of more immediate medical interest.

